# Evaluation of Acetabular Defects in Children with Cerebral Palsy: A Comparative Analysis of CT Measurements and Radiographic Parameters

**DOI:** 10.3390/children12091254

**Published:** 2025-09-17

**Authors:** Domenic Grisch, Olivier Weber, Britta K. Krautwurst, Franziska L. Hatt, Michael Zellner, Christian von Deimling, Tobias Götschi, Bastian Sigrist, Thomas Dreher

**Affiliations:** 1 Pediatric Orthopedics and Traumatology, University Children’s Hospital Zurich, 8008 Zurich, Switzerlandbritta.krautwurst@kispi.uzh.ch (B.K.K.); franziska.hatt@kispi.uzh.ch (F.L.H.);; 2 Department of Diagnostic Imaging, University Children’s Hospital Zurich, 8008 Zurich, Switzerland; michael.zellner@kispi.uzh.ch; 3 Children’s Research Centre, University Children’s Hospital Zurich, 8008 Zurich, Switzerland; 4 Clinical Trials Unit, University Hospital Balgrist, University of Zurich, 8008 Zurich, Switzerland; tobias.goetschi@balgrist.ch; 5 Research in Orthopedic Computer Science (ROCS), University Hospital Balgrist, University of Zurich, 8008 Zurich, Switzerland; 6 Department of Pediatric Orthopedics, Balgrist University Hospital Zurich, University of Zurich, 8008 Zurich, Switzerland

**Keywords:** cerebral palsy, acetabular morphology, radiographic measurement techniques

## Abstract

**Highlights:**

**What are the main findings?**

- Three-dimensional CT measurement technique is an important addition to the conventional radiographic measurements.
- Conventional radiographic measurements demonstrated better discriminative power for identifying hip dislocation and correlated strongly with the 3DAI (*p* < 0.001).

**What is the implication of the main finding?**

- More precise quantification of the defect amount and direction of hip dislocation via 3DAI method
- Improvement of operative planning for better postoperative outcome

**Abstract:**

**Objectives**: This retrospective study examines acetabular morphology and defects in children with cerebral palsy (CP). The study discovers the usefulness and reliability of a reconstructed 3D CT measurement technique and compares it to conventional radiographic measurements. **Methods**: 33 subjects with CP who underwent hip reconstruction, including Dega osteotomy and varus derotation femoral osteotomy, were included and compared to an age-matched group of 42 typically developing children. We reproduced a three directional acetabular index (3DAI), including anterosuperior, superolateral and posterosuperior indices in CT analysis, and compared them with established radiographic measurements for the migration percentage (MP) and the acetabular index (AI). **Results**: The results showed significantly higher 3DAI in every direction of wall deficiency, accentuating the methods sensitivity for acetabular dysplasia. The interrater and test–retest reliability were robust with ICC = 0.939–0.988 for the CP group. Conventional radiographic measurements demonstrated better discriminative power for identifying hip dislocation and correlated strongly with the 3DAI (*p* < 0.001). **Conclusions**: The 3DAI method showcases an important addition to the conventional radiographic measurements by enabling a quantification of the defect amount and direction for operative planning. The study supports the potential of a 3D analysis in the improvement of diagnostic precision and suggests a continuous refinement of the CT measurement technique.

## 1. Introduction

Cerebral palsy (CP) is a complex disorder that affects an individual’s ability to move due to spastic muscle contraction, stemming from brain-related issues during fetal or early infant stages [1,2,3]. Hip dislocation accompanied by acetabular dysplasia is a common finding in children with CP and a higher severity of motor dysfunction [4,5]. Non-ambulatory children present the highest incidence rates up to 90% [6]. In CP, femoral head subluxation is described as typically posterosuperior, aligning with acetabular deficiency [7]. Since most subluxations are described as posterosuperior (37%), there are still 29% anterior and 15% mid-superior or mixed [8]. It is known that major acetabular deficiency coincided with the direction of the subluxation or dislocation. Even mild cases of CP show posterior wall deficiency compared to normal controls, progressing to anterosuperior and superolateral quadrants with disease severity [7]. Therefore, a precise analysis of the acetabular deficiency and the correlating direction of subluxation or dislocation is necessary to allow accurate planning of the surgical treatment. Often, early hip reconstruction including pelvic osteotomy to restore acetabular shape and femoral corrective osteotomy is necessary to avoid pain, loss of functions and limitations of important basic functions [9,10]. The recurrence rates after reconstruction, however, are reported to be up to 23% [11] and the risk of perioperative morbidity is enormous with 63% [12].

Due to an abnormal development of the hip, the acetabulum matures insufficiently, and the femoral head is not held stable in its socket. The acetabular deficit, as well as its position and the direction of the dislocation, are usually assessed using an anteroposterior pelvic radiograph. For this, the Migration Percentage (MP) and the Acetabular Index (AI) are determined [13]. This is an objective but only 2D assessment, which brings the accuracy into question. Hip migration in cerebral palsy, however, is a three-dimensional problem. Therefore, it is necessary to implement a 3D computer tomography (3D-CT) assessment to understand hip migration accurately.

Several studies have used 2D sectional planes to reconstruct a 3D space for a more accurate analysis of acetabular dysplasia in CP [6,8,14]. To increase the precision of the acetabular defect measurement, Chung et al. utilized multiplanar reformation techniques to create the three-directional acetabular index (3DAI), consisting of an anterosuperior, superolateral and posterosuperior index. The anterosuperior index dissecting the anteroinferior iliac spine and the posterior end of the ischial tuberosity on the true lateral image. The superolateral index is dissecting the center of the acetabulum parallel to the line connecting the anterosuperior iliac spine and the symphysis. Last, the posterosuperior index measured in the plane which dissected the line of the symphysis pubis and the posteroinferior iliac spine. The three indices represent all three planes, but the individual indices are measured using 2D sectional images in the CT. As another limitation of the study, the number of hips included is small and the comparison to a reference group does not take age into account [15].

An assessment of possible developmental deviations requires a reference group. This cohort should be as similar as possible, emphasizing the need for age matching, which was absent from the previous investigations. The primary aim of this study is not only to compare visual defect localization with the 3DAI but to compare the three-dimensional morphology of children with typically developing children. The study should help to understand the 3D anatomy of children with CP where there is an indication for hip reconstruction. The second aim was to evaluate whether the 3D indices according to Chung et al. correlate with the conventional radiographic measurements and to evaluate which method is suited for the assessment of hip dislocation tendencies and stability [7]. This is the first study, comparing the findings of measurement techniques in CP patients to a healthy control group.

## 2. Materials and Methods

### 2.1. Patients

For retrospective analysis, we included 33 children (18 boys, 15 girls) with uni- or bilateral CP to analyze the hip morphology, who had undergone corrective Dega osteotomy of the acetabulum, combined with varus derotation femoral osteotomy. Inclusion required a preoperative X-ray and CT scan with a maximum cutting thickness of 2 mm and existing consent between 2006 and 2022. Unilateral involvement was found in only one case, with the unaffected side being excluded. For age comparison, three age groups were defined: children aged 4–6 (*n* = 17), 7–9 (*n* = 28) and 10–14 (*n* = 20).

The ambulatory ability was graded by the Gross Motor Function Classification System (GMFCS) [5]. Children with secondary neuromuscular diagnoses were excluded. An age-matched control group was formed from 40 typically developing children with CT scans of the abdomen, pelvis, or lower limbs, in which the indications for the CT were general trauma not including hip, pelvis and femur or underlying disease not affecting the measured hip. In case of unilateral, independent hip problems, the unaffected hip was included (e.g., unilateral Perthes disease or cystic changes in the femoral bone structure). Children with underlying illnesses affecting the musculoskeletal system and mobility were excluded. Since the performed CT scan was carried out closely after injury, we do not expect any trauma-related musculoskeletal or anatomical changes affecting the hip due to trauma if they were not reported before. All children were at least 4 years old and showed open triradiate cartilage. After excluding any affected hips, 67 typically developed hips remained.

All participants had given their consent to the further use of the data for scientific purposes. The cantonal ethics committee approved the project (BASEC-Nr. 2023-00363).

### 2.2. CT Measuring

CT scans were carried out using a multidetector CT scanner with low-dose settings and a slice thickness of 0.625 mm. All CT scans performed in-house followed the ALARA principle (as low as reasonably achievable) using comparable low-dose protocols. Different CT scanners were used during the study period (2006–2022). From 2006 to 2013, examinations were conducted on a Philips Brilliance 40 (Philips Healthcare, Best, The Netherlands). Between 2013 and 2019, a Philips Brilliance 64 (Philips Healthcare, Best, The Netherlands) was used. Starting in 2019, scans were acquired with a GE Revolution (GE Healthcare, Chicago, IL, USA). The routine CT scans of the pelvis performed in children with CP from 2019 onward were carried out with a mean dose length product (DLP) of 10.22 mGy*cm and a mean computed tomography dose index volume (CTDIvol) of 0.52 mGy. In comparison, scans performed before 2019 showed considerably higher values, with a mean DLP of 31.01 mGy*cm and a CTDIvol of 1.26 mGy. The scan range was defined as the area from just above the anterosuperior iliac spine to just below the ischial tuberosity. The CT scan is already a clinical standard method for the evaluation of hip dislocation prior to surgery. An MRI would show the chondral coverage of the hip more precisely, but it would require anesthesia for examination. We used retrospective, already-performed CT scans for our analysis.

**Figure 1:**
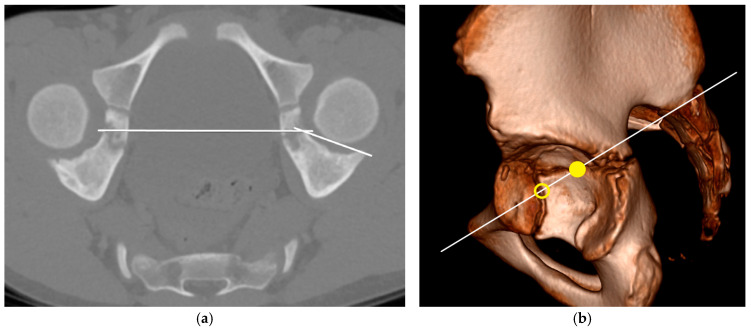
(**a**) Reformatted CT scan for the measurement of the PSI, (**b**) Plane of the PSI with intersection of the triradiate cartilage in the posterior (point) and inferior limb (circle).

For the objective assessment of acetabular defects in the CT scans, we used the 3DAI method introduced by Chung et al. [15]. This method consists of three measurements: the anterosuperior index (ASI), the superolateral index (SLI) and the posterosuperior index (PSI). To obtain these values, we generated oblique plane images through multiplanar reformation. The measurements were conducted simultaneously with the description by Chung. When forming the plane for the PSI, both the inferior and posterior limbs of the triradiate cartilage are displayed. Contrary to Chung’s description, we decided to use the posterior limb and not the inferior limb as the coordinate for evaluation. In our anatomical understanding, the posterior limb forms the lower boundary of the acetabular roof formed by the ilium (Figure 1).

### 2.3. Visual Assessment

**Figure 2:**
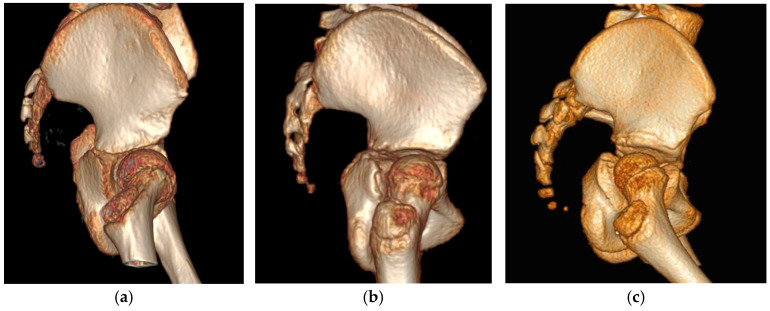
Visual assessment of different acetabular defects: (**a**) anterior, (**b**) global and (**c**) posterior.

For the visual assessment of the acetabular defect, two presentations (PowerPoint software, Microsoft, Redmond, Washington) were compiled by an independent person, each containing screenshots of all included 3D reconstructed hips from the CP group, presented in a random order. These presentations included lateral and frontal views of the hips, including the femoral head. Hips were categorized into three groups: the subluxation group, the dislocation group, and the stable group. Regarding the location of acetabular defects, the increased steepness of the acetabulum was classified as either anterior, global (mixed) or posterior (Figure 2).

### 2.4. Radiographic Evaluation

According to clinical routine, the AI and MP as defined by Reimers were used to assess the acetabular development and the centering of the hip in the CP group using anteroposterior pelvic radiography [16]. Referring to the clinical standard, we used the modified index by Reimers and used the middle of the sourcil for the lateral aspect of the acetabulum. The acetabular sourcil is characterized by increased sclerosis with a concave shape and refers to the lateral boarder of the weight-bearing area of the acetabulum. The AI utilizes the sclerotic zone as the lateral aspect of the acetabulum. The time interval between the radiographs and the CT scan was defined as less than three months.

### 2.5. Data Analysis

To assess the congruence in the CP group between the acetabular defect direction analyzed with the 3DAI method and the visual impression of the defect in the reconstructed 3D-CT, we compared the result of the 3DAI with the visually analyzed defect directions. Moreover, we explored the correlation between the 3DAI values and the degree of dislocation by comparing them to the visually evaluated hip-centering. The imaging measurements are interval-scaled in degrees, while the subjective evaluation was expressed as a categorical variable.

To evaluate the hip centering visually, we utilized two presentations with the same 3D-reconstructed CT images of all hips of the CP group in different orders. The medical master’s degree student and the researcher were both instructed prior to the experiment by the surgeons for assessment of acetabular deficiencies. The presentations were displayed on the identical computer monitors (HP EliteDisplay E243, 23.8 inch, HP, Palo Alto, CA, USA), with the same settings for each examiner. Two examination sessions were conducted, with two weeks in between. Patient information was not disclosed to any of the examiners.

For investigation of 3DAI, we formed an age-matched reference group and compared them to the CP group.

Inter-rater and test–retest reliability were determined for each index to demonstrate the measurement accuracy of 3DAI. For this purpose, 21 hips from the CP group and 22 hips from the reference group were randomly selected by an independent person. A team consisting of pediatric orthopedic surgeons with 17 and 7.5 years of experience, a medical master’s degree student and a researcher determined the 3DAI once for these hips, except for the master’s degree student, who carried out the measurements three times. To set the landmarks correctly, the four people underwent a structured training session before measuring, during which two representative examples were used to demonstrate correct landmark placement. In case of conspicuously misplaced landmarks, the person was re-examined, and all landmarks were subsequently reassigned. Based on the repeated measurements, we calculated intra- and inter-rater variability to evaluate the robustness and reproducibility of this method.

Last, we correlated the 3DAI with the conventional measurement method, the standard X-rays. The X-rays were evaluated in the CP group during the same period as the CT scans. Our intention was to determine if there is a correlation between the conventional radiographic measurement techniques and the 3DAI. Hence, we assessed a correlation between the 3DAI values and the MP and AI and compared them to the visually assessed dislocation levels.

### 2.6. Statistical Analysis

Descriptive statistics are presented as the mean and standard deviation or relative frequencies as applicable. Subject characteristics were summarized on a per hip basis. In subjects where both hips were included in the study, subject-based parameters were hence counted twice. The inter-rater and test–retest reliability of interval-scaled measurements were quantified in terms of the intraclass correlation coefficient (ICC (2,1)) [17], based on a two-way random effects model assessing the absolute agreement of a single-measure approach and the related standard error of measurement [18]. ICC values were classified as poor (ICC ≤ 0.2), fair (0.2 < ICC ≤ 0.4), moderate (0.4 < ICC ≤ 0.6), good (0.6 < ICC ≤ 0.8) and very good (0.8 < ICC) [19]. Inter-rater and test–retest agreement of categorical scores were quantified using Krippendorff’s α, which was classified as poor (α ≤ 0.5), moderate (0.5 < α ≤ 0.67), good (0.67 < α ≤ 0.8) or excellent (0.8 < α) [20]. No gross deviations from normality were observed and parametric models were utilized. To account for side-to-side dependencies within patient measurements, we employed linear mixed effects models (diagonal covariance structure, restricted maximum likelihood), with side as a random factor, followed by post hoc pairwise comparisons (Bonferroni corrected). Frequency distributions among groups were assessed for statistical significance with Fisher’s exact tests. Simple pairwise comparisons were performed with paired t-tests. The discriminative capacity of radiograph and CT-based anatomical measurements in predicting hip instability was quantified by their receiver operator characteristics (ROC) and their associated area under the curve (AUC). Further, we analyzed the predictive performance at each predictor’s most efficient cutoff (Youden index). For the prediction of subluxation, cases suffering from dislocation were excluded, whereas all cases were included in the prediction of dislocation. The analysis was conducted with SPSS (Version 28.0. Armonk, NY, USA: IBM Corp) and MATLAB (The MathWorks Inc. (2022b), Natick, MA, USA). *p*-values below 0.05 were considered statistically significant.

## 3. Results

A total of 65 hips from the CP group and 67 from the reference group were evaluated. The mean age of the children in the CP group at the time of CT scan was 8.6 years (SD 3.1) and in the control group, 9.3 years (SD 3.2). The CP group were 53.8% male, and the reference group 70%. Most of the children with CP had GMFCS V (52.3%) or IV (35.4%), while GMFCS III (9.2%) and II (3.1%) were rare. There was no child with GMFCS I. In 72.3% of the CT scans in the CP group and 70% in the reference group, the slice thickness was 1 mm and lower. None of the scans exceeded a thickness of 2 mm.

The evaluation of the 3DAI revealed significantly higher values in all the Chung Indices within every wall deficiency group (global, posterior, and anterior) compared to the control group (in all comparisons *p* < 0.001 (see Table S1), except for PSI anterior vs. control *p* = 0.048), (Table 1). We observed that the index aligned with the visually defined acetabular defect exhibited the greatest deviation from the norm in pairwise comparison. Furthermore, the three indices increased across the stable, subluxated and luxated groups with the degree of luxation (*p* < 0.001), (Table 2).

Test–retest and inter-rater reliability for the 3DAI were good to very good and more favorable in the CP group (ICC = 0.939–0.988) compared to the reference group (0.679–0.970). The visual assessment, in comparison, showed a poor to moderate reliability (α = 0.459–0.641) in the inter-rater evaluation between all raters, and a good to excellent performance (α = 00.745–0.813) between the clinical experts. This shows that quantitative measuring of the 3DAI indices performed overall better in the inter-rater comparison than the visually evaluated hip centering. All three 3DAI showed increased standard deviations (SD) in the CP group, reflecting variability in dysplasia (Table 1).

In the CP cohort, the ASI was found to be significantly higher among the oldest age group compared to the younger age groups (*p* = 0.005), while no such correlation was found for the SLI and the PSI (*p* = 0.264 and *p* = 0.103), (Table 3). No significant association between 3DAI and age was found in the control group (Table 3). In the visual evaluation, age differed significantly among the three categories of wall deficiencies (*p* = 0.020), (Table 4), while pairwise comparison showed that primarily, the posterior wall defects showed a younger mean age than the global defects (*p* = 0.006). No statistically significant age differences were found among the stable, subluxation, and dislocation groups (Table 4).

Furthermore, we found that regardless of the direction of the wall defect, the frequency of clinically assessed occurrences of luxation and subluxation remained the same (*p* = 0.335).

**Figure 3:**
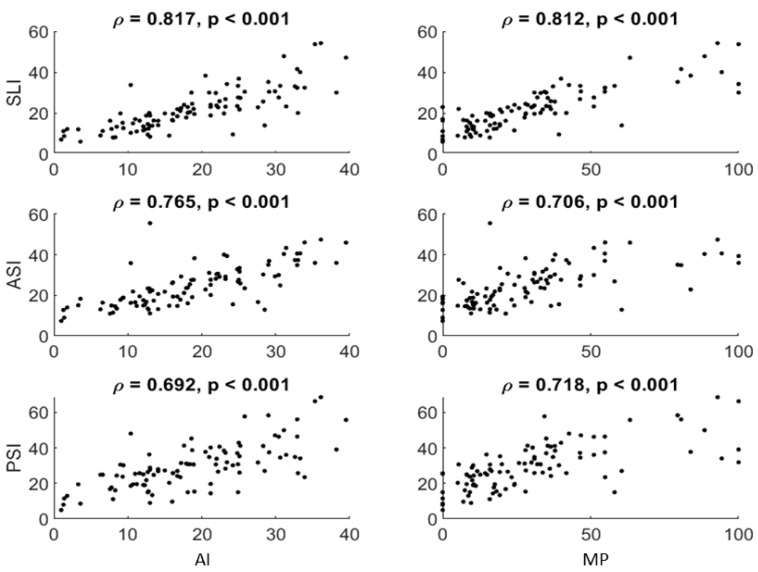
Scatterplots of associations between 2D (x-axis) and 3D (y-axis) measurements. *p*: Spearman rank correlation coefficient.

**Figure 4:**
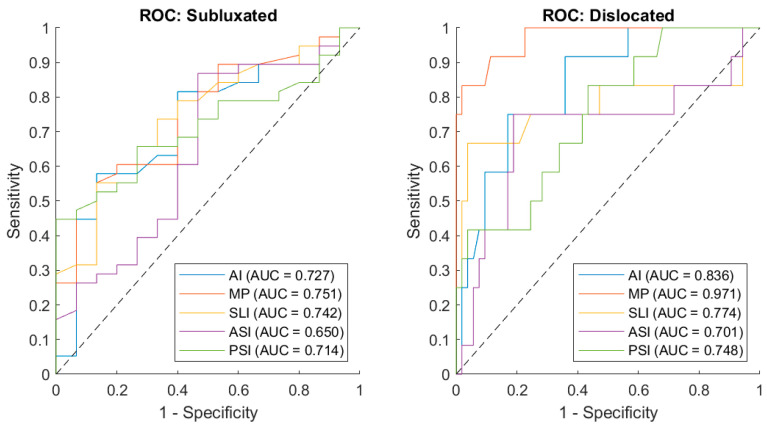
ROC curve of the two radiograph-based indices (AI, MP) and the three CT-based indices (SLI, ASI, PSI).

Evaluating the 3DAI method to conventional radiographic measurements, we identified a high correlation between all three 3DAI and the conventional radiographic metrics MP and AI (all *p* < 0.001), with the SLI displaying the strongest correlation (Figure 3). ROC analysis indicated that MP possessed the best discriminative performance in distinguishing both subluxated and dislocated cases from stable hips with a high to very high diagnostic precision (subluxated group AUC = 0.751 and dislocated group AUC = 0.971) (Figure 4). The 3DAI and the AI performed comparably well (AUC = 0.650–0.836). The MP shows the best predictability in dislocated hips (sensitivity 83.30% and specificity 98.10%) (Table 5).

## 4. Discussion

The current gold standard in the assessment of acetabular dysplasia and associated hip joint displacements relies on 2D radiographic images. Distinguishing between anterior, posterior, lateral and mixed deficits in the coverage of the acetabulum is relevant for surgical correction in cerebral palsy (CP) [7]. The anterior and posterior femoral head dislocation is not considered by measurements such as MP and AI in radiographs, which only evaluate lateral bone coverage [21]. Furthermore, measurement is influenced by pelvic obliquity, tilt and/or rotation, which is a common problem in CP patients, particularly in measurements that depend on Hilgenreiner’s line [22]. Moreover, poor quality of radiographs may impact the accuracy, which may influence therapeutic choices [22,23], given the fact that an MP exceeding 40% is the generally accepted threshold for surgical intervention [24]. Additionally, the effects of hip displacement and the association of GMFCS level on acetabular dysplasia highlights the need for three-dimensional evaluation in children with higher GMFCS levels [7].

Additionally, for a 2D radiograph, a 3D CT scan is already clinical standard prior to surgery in selected centers. These centers use pre-operative 3D CT for planning and understanding the acetabular deficiency. The results of our study underline the importance of having 3D data available. The value of an additional diagnostic method is high if it provides more new information, which is crucial for understanding a problem and to improve the planning for its treatment. Until now, no precise measurement techniques are implemented to gain information about the exact hip positioning through this CT scan. Different investigations focused on the three-dimensional description of the acetabular dysplasia. Chung et al. introduced a 3DAI method describing a three-dimensional space with sectional planes. This method is still a 2D analysis relying on 2D CT scans and is missing a three-dimensional description to achieve precise preoperative analysis of the morphology [15]. This raises two concerns: First, anatomically defined points in the three-dimensional space of the hip must be translated into a two-dimensional image, introducing flexibility into determining the sectional plane. Second, manual adjustments for pelvic obliquity are required, further contributing to the introduced flexibility.

Also, Chung et al. did not perform age-matching; there are no normative reference values for different age groups.

Considering that different CT-based studies have already been performed, we expected to replicate some of their results. Chung et al. reported increased 3DAI angles in the direction of wall defects and a correlation between 3DAI, GMFCS level and MP [7,15]. Acetabular defects in CP have been reported as anterior, posterior, mid-superior and mixed, with an overall preference for the posterior region [6,8]. Moreover, prior CT studies have shown a correlation between anterior acetabular defects and increasing MP [10,12], implying a potential correlation between ASI and MP. We also anticipated a correlation between the SLI in the CT and the AI in the X-ray, since both screen for lateral acetabular wall deficiency.

Our findings corroborate the findings reported by Chung et al., who observed increased 3DAI angles in the direction of wall defects. Furthermore, all indices exhibited significantly higher values compared to the control group, regardless of the region (anterior, posterior, global) where the visually assessed defect was located. This leads us to the conclusion that there is probably a general deviation from the norm in acetabular development of children with CP with either anterior or posterior migration. The increase in 3DAI indices among the luxation groups indicates that higher 3DAI is associated with a higher grade of dysplasia.

In age comparison, we discovered that the ASI mainly showed statistically significant higher values in the oldest age group compared to the two younger age groups, while the SLI and PSI showed no such correlations. The visual evaluation that was performed in our study showed that younger children mainly presented posterior deficiencies, while the older age groups had more combined posterior and anterior defects. Hence, the visual assessment on a 3D CT is lacking in precision compared to the objective assessment and does not recognize all anterior defects, especially because pelvic tilt may lead to overestimation of anterior coverage.

The inter-rater reliability showed better results for 3DAI measuring than for visual assessment between non-clinical experts, suggesting that this is less dependent on observer’s experience. Additionally inter-rater and test–retest reliability demonstrated better performance in the CP group compared to the reference group. This can be attributed to the larger standard deviation (SD) in the CP group, due to differences in the degree of dysplasia. The higher overall values in the CP group means that trivial differences in measurements carry less statistical weight compared to the reference group, whereas, with a small SD, as found in the reference group, even minor differences in measurements can lead to a statistical, but not clinically relevant, effect.

Consistent with our assumptions based on earlier research, the findings showed relationships between 3DAI, MP, and AI. The strongest correlation between radiographic measurements and 3DAI indices was found for the SLI. Since the X-ray is an overlaying image, it might present the lateral aspect of the acetabulum best. Also, SLI presents the roof of the acetabulum and larger roof defects will be more likely to be detected in a planar X-ray by MP and AI. MP and AI were still found to be better in differentiating between hips of children with cerebral palsy and typically developing hips in terms of distinguishing between stable, subluxated and luxated hips, showing their further usefulness for the indication of operative corrections. However, the 3DAI is a valuable tool for surgical planning through quantifying the direction and size of the defect and considering this during the acetabular reconstruction procedure. Furthermore, in low-dose technique, it may serve as a follow-up parameter to assess the effectiveness of a correction and to detect recurrent acetabular deficiency.

The main limitation of our study lies within measurement precision, which is influenced by various factors such as image quality, age, examiners’ experience and the 3DAI method itself. The 3DAI technique is an indirect 3D examination of the hip, still measuring different planes in a 2D image of the 3D examination. Lower image quality leads to imprecision in the placement and following measurement of the sectional plane. With children’s age, their epiphyseal plates narrow; this leads to a challenge in correctly placing the measurement lines. Additionally, the expertise of the examiners to set the correct planes for angle measurement is crucial and a certain amount of training leads to a higher accuracy. Additionally, not only is the bony acetabulum crucial for femoral head coverage, but also the acetabular cartilage that leads to good femoral head position. The CT scan, however, does not show cartilage coverage of the femoral head, and can lead to over diagnosed acetabular deficiency. The disadvantage of this measuring technique is the necessity of a CT scan. The measurements are limited to the availability of a CT scan. Selected centers use pre-operative 3D CT for planning and understanding acetabular deficiency. For centers who use 3D CT for planning, there are not additional costs and efforts for the patients and families. Additional CTs in other centers create more costs and some more radiation for the patients, however we believe that the benefit of attaining important information for surgical planning and reduction in potential risks of not addressing the acetabular deficiency will outweigh the increased efforts for the CT. Unfortunately, in our study the number of different age groups was still too small to provide age-stratified reference ranges for ASI, SLI and PSI. Only a comparison with the available data from typically developing children was possible. For future planning, the amount of dislocation is another important factor to consider in a 3D MP, to integrate into surgical planning.

Future studies should be carried out to address these limitations of the routine radiological analysis (X-ray and CT). Current work is going on around the creation of a 3D model from the CT scan and measuring the planes in the 3D model. This can increase the measurement accuracy and gives us a more precise description of the hip dislocation and wall deficiency.

## 5. Conclusions

In conclusion, our study highlights the great potential of three-dimensional analysis in providing a comprehensive evaluation of acetabular defects in children with CP. The 3DAI method tries to address the limitations of common uniplanar radiographic methods in interpretation of the acetabular deficiency by considering anterior, lateral and posterior bone coverage. This three-dimensional information is crucial for the understanding of acetabular deficiency and should be used for planning of surgical acetabular reconstruction. However, future studies and a larger data collection of typically developed hips are necessary to support the understanding of the anatomy and its development, and to create a normative database for each age group. Furthermore, an artificial intelligence AI-supported algorithm may be used to standardize dysplasia evaluation and help clinicians to develop 3D treatment plans pre-operatively.

## Figures and Tables

**Table 1:** Three directional acetabular indices (3DAI) in cerebral palsy (CP) vs. control group (CG) in relation to subjective defect assessment. All values are given in degrees. SLI = superolateral index, PSI = posterosuperior index, ASI = anterosuperior index, SD = standard deviation.

Degree of Dislocation	SLI		ASI		PSI	
	Mean	SD	Mean	SD	Mean	SD
Dislocation	35.1	14.2	33.7	10.9	44.6	14.6
Subluxation	25.3	7.4	29.0	9.3	33.4	12.0
Stable	20.2	5.7	23.1	8.4	26.3	8.2
Control	13.1	3.6	17.4	3.9	20.3	7.4

**Table 2:** Dependence of 3DAI on the degree of luxation in CP children. All values are given in degrees. CP (cerebral palsy), 3 DAI (three directional acetabular indices), SLI (superolateral index), PSI (posterosuperior index), ASI (anterosuperior index), SD (standard deviation).

Wall Deficiency	SLI		ASI		PSI	
	Mean	SD	Mean	SD	Mean	SD
Global	26.1	7.4	30.3	9.3	34.1	7.5
Posterior	29.5	11.0	27.5	9.1	39.1	13.0
Anterior	21.3	8.5	28.3	11.6	26.9	13.9
Control	12.9	3.6	17.3	3.9	20.2	7.4

**Table 3:** Dependence of age on the 3DAI of CP children and control group. All values are given in degrees. CP (cerebral palsy), 3 DAI (three directional acetabular indices), SLI (superolateral index), PSI (posterosuperior index), ASI (anterosuperior index), SD (standard deviation).

	Age Groups					
	4–6 Years		7–9 Years		10–14 Years	
	Mean	SD	Mean	SD	Mean	SD
SLI-CP	26.8	8.2	27.1	11.6	23.6	8.6
SLI-Control	15.9	3.8	12.1	3.7	12.3	2.5
ASI-CP	27.0	7.7	26.6	9.1	32.3	11.8
ASI-Control	19.3	4.2	16.7	3.7	16.8	3.8
PSI-CP	38.2	12.9	34.2	14.9	29.6	9.0
PSI-Control	23.4	6.5	20.7	6.7	18.1	7.9

**Table 4:** Age dependence on defect localization and degree of luxation in CP children. All values provided represent age numbers. CP (cerebral palsy), SD (standard deviation).

		Age	
		Mean	SD
Wall Deficiency	Anterior	8.9	2.2
	Global (mixed)	10.0	3.5
	Posterior	7.4	3.1
Degree of Dislocation	Dislocation	10.0	3.5
	Stable	9.1	3.0
	Subluxation	7.9	2.9

**Table 5:** Cut-off values for ROC analysis. Sensitivity and specificity for different parameters.

Group	Predictor	Cutoff	Sensitivity	Specificity	Accuracy
Subluxated	AI	22.2	57.90%	86.70%	66.00%
	MP	33.8	55.30%	86.70%	64.20%
	SLI	24	55.30%	86.70%	64.20%
	ASI	21.2	86.80%	53.30%	77.40%
	PSI	37.2	44.70%	100.00%	60.40%
Dislocated	AI	28.5	75.00%	83.00%	81.50%
	MP	60.5	83.30%	98.10%	95.40%
	SLI	34.2	66.70%	96.20%	90.80%
	ASI	34.9	75.00%	81.10%	80.00%
	PSI	31.9	83.30%	56.60%	61.50%

## Data Availability

The data presented in this study are available on request from the corresponding author due to privacy reasons of the included patients. All data is stored at the University Children’s Hospital, Zurich.

## Supplementary Materials

The following supporting information can be downloaded at: https://www.mdpi.com/article/10.3390/children12091254/s1, Table S1: 95% CI intervals of the three-dimensional indices SLI, ASI and PSI.

## Abbreviations

The following abbreviations are used in this manuscript:

CP	Cerebral Palsy
3DAI	Three Directional Acetabular Index
MP	Migration Percentage
AI	Acetabular Index
3D-CT	3D computer tomography
GMFCS	Gross Motor Function Classification System
ASI	Anterosuperior Index
SLI	Superolateral Index
PSI	Posterosuperior Index
ICC	Intraclass correlation coefficient
SEm	Standard error of measurement
ROC	Receiver operator characteristics
AUC	Associated area under the curve
