# Supplementary material for: Evaluation of Acetabular Defects in Children with Cerebral Palsy: A Comparative Analysis of CT Measurements and Radiographic Parameters
Source: Children (Basel). 2025 Sep 17;12(9):1254. doi: 10.3390/children12091254 (PMC12468863; doi:10.3390/children12091254)
Supplement: Supplementary file 1 [file children-12-01254-s001.zip › children-3822345-supplementary.pdf]

Supplementary Materials

Table S1. 95% CI intervals of the three-dimensional indices SLI, ASI and PSI.

		SLI			ASI			PSI		
		**p–Value**	**95% CI**		**p–Value**	**95% CI**		**p–Value**	**95% CI**	
			**Lower Bound**	**Upper Bound**		**Lower Bound**	**Upper Bound**		**Lower Bound**	**Upper Bound**
Control	Global	<0.001	–18.200	–7.996	<0.001	–18.446	–7.525	<0.001	–21.177	–6.667
	Posterior	<0.001	–20.748	–12.182	<0.001	–14.773	–5.606	<0.001	–25.020	–12.840
	Anterior	<0.001	–12.943	–3.546	<0.001	–16.002	–5.944	0.048	–13.399	–0.036
